# Supplementary material for: Medicare Advantage in Soft Tissue Sarcoma May Be Associated with Worse Patient Outcomes
Source: J Clin Med. 2023 Aug 4;12(15):5122. doi: 10.3390/jcm12155122 (PMC10420157; doi:10.3390/jcm12155122)
Supplement: Supplementary file 1 [file jcm-12-05122-s001.zip › jcm-2450073-supplementary.pdf]

Journal of
Clinical Medicine

MDPI

Supplemental Table S1. ICD-10 and CPT Codes

ICD-10 Codes	Description
Soft Tissue Excision (Lower Extremity)	0KBN-, 0KBP-, 0KBQ-, 0KBR-, 0KBS-, 0KBT-, 0KBV-, 0KBW-, 0JBL-, 0JBM-, 0JBN-, 0JBP-, 0JBQ-, 0JBR-, 0LBL-, 0LBM-, 0LBN-, 0LBP-, 0LBQ-, 0LBR-, 0LBS-, 0LBT-, 0LBV-, 0LBW-, 0MBL-, 0MBM-, 0MBN-, 0MBP-, 0MBQ-, 0MBR-, 0MBS-, 0MBT-, 0MBV-, 0MBW-,
Soft Tissue Resection (Lower Extremity)	0KTN-, 0KTP-, 0KTQ-, 0KTR-, 0KTS-, 0KTT-, 0KTV-, 0KTW-, 0LTJ-, 0LTK-, 0LTL-, 0LTM-, 0LTN-, 0LTP-, 0LTQ-, 0LTR-, 0LTS-, 0LTT-, 0LTV-, 0LTW-, 0MTL-, 0MTM-, 0MTN-, 0MTP-, 0MTQ-, 0MTR-, 0MTS-, 0MTT-, 0MTV-, 0MTW-,
Soft Tissue Sarcoma (Lower Extremity)	C49.20, C49.21, C49.22
**CPT Codes**	**Description**
Soft Tissue Excision (Lower Extremity)	27047, 27043, 27048, 27045, 27327, 27337, 27328, 27339, 27618, 27632, 27619, 27634, 28043, 28039, 28045, 28041
Soft Tissue Resection (Lower Extremity)	27049, 27059, 27329, 27364, 27615, 27616, 28046, 28047
